# Impact of mental health problems on case fatality in male cancer patients

**DOI:** 10.1038/bjc.2012.150

**Published:** 2012-04-19

**Authors:** G D Batty, E Whitley, C R Gale, D Osborn, P Tynelius, F Rasmussen

**Affiliations:** 1 Department of Epidemiology and Public Health, University College London, London, UK; 2 Centre for Cognitive Ageing and Cognitive Epidemiology, University of Edinburgh, Edinburgh, UK; 3 Medical Research Council Epidemiology Resource Centre, University of Southampton, Southampton, UK; 4 Mental Health Sciences Unit, University College London, London, UK; 5 Child and Adolescent Public Health Epidemiology Group, Department of Public Health Sciences, Karolinska Institute, Stockholm SE-17176, Sweden

**Keywords:** mental health, mortality, survival, cohort

## Abstract

**Background:**

Although mortality rates are elevated in psychiatric patients relative to their healthy counterparts, little is known about the impact of mental health on survival in people with cancer.

**Methods and results:**

Among 16 498 Swedish men with cancer, survival was worse in those with a history of psychiatric hospital admissions: multiply-adjusted hazard ratio (95% confidence interval) comparing cancer mortality in men with and without psychiatric admissions: 1.59 (1.39, 1.83).

**Conclusion:**

Survival in cancer patients is worse among those with a history of psychiatric disease. The mechanisms underlying this association should be further explored.

Mental health problems are of major public health importance and exact considerable health care expenditure (Kessler *et al*, 2005; Eaton *et al*, 2008). Individuals with mental health problems have less healthy lifestyles than the general population, for example, they are more likely to smoke (de Leon and Diaz, 2005), drink heavily (Davidson *et al*, 2001), have a poor diet (Davidson *et al*, 2001; McCreadie, 2003), and exercise less (Daumit *et al*, 2005). In addition, psychiatric patients may have worse access to health care (Cradock-O'Leary *et al*, 2002), be less likely to take part in screening programmes (Howard *et al*, 2010), and psychiatric staff, with whom they have most contact, may be less efficient at diagnosing physical problems, particularly in the context of psychiatric comorbidities (Rigby and Oswald, 1987). It is therefore perhaps unsurprising that individuals with mental health problems appear to have shorter life expectancy than their healthy counterparts (Harris and Barraclough, 1998; Eaton *et al*, 2008; Lawrence *et al*, 2010).

Results from an extensive literature on psychiatric illness and cancer mortality are equivocal, with individual studies reporting increased, similar, or decreased risk of cancer mortality in psychiatric patients (Lawrence *et al*, 2010). In interpreting these results, it is worth noting that cancer mortality is a combination of cancer incidence, which has been widely explored, and survival/case fatality, which remains poorly understood in this context. One recent study reported lower 1- and 5-year cancer survival in individuals with schizophrenia or other psychosis (Dalton *et al*, 2008). Another noted that cancer mortality rates in psychiatric patients were higher than cancer incidence rates, prompting speculation that case fatality might be greater in this group (Kisely *et al*, 2008). However, we are only aware of one study (Lawrence *et al*, 2000) that has directly examined cancer case fatality in psychiatric patients. Accordingly, we report on psychiatric disorder–case fatality associations in a large, complete, birth cohort of male Swedish cancer patients.

## Materials and methods

The record linkage used to generate this cohort has been reported previously (Batty *et al*, 2007). All non-adopted men born in Sweden from 1950–1976 with both biological parents identified in the Multi-Generation Register were identified and linked to cause of death, cancer, and National Hospital Discharge Registers. Study approval was obtained from the Regional Ethics Committee, Stockholm. We identified all men with (non-fatal) cancer registrations at age 18+ from the Swedish Cancer Register. Men with mental health disorders were identified from the hospital admissions data from the Swedish Hospital Discharge Register.

Cancer case fatality was compared in men with and without psychiatric hospital admissions. Survival times from first cancer registration to cancer death in men with and without a psychiatric hospital admission were compared using log-rank tests and Cox (proportional hazards) regression. In the main analyses, we excluded men whose cancer registration pre-dated their first psychiatric admission to allow for the possibility of reverse causality, that is, less favourable cancer prognosis leading to a psychiatric admission rather than the converse. This approach may be overly conservative (Whitley *et al*, 2012) and so analyses were repeated with these men included.

## Results

Analyses are based on 16 498 men with cancer registrations aged 18+ years. The majority of men (*N*=13 940 (84.5%)) had a cancer considered to be unrelated to smoking and around a sixth (*N*=2558 (15.5%)) had a smoking-related cancer (lung, oral cavity, nasopharynx, oropharynx, hypopharynx, nasal cavity, and paranasal sinuses, larynx, oesophagus, stomach, pancreas, liver, kidney (body and pelvis), ureter, urinary bladder, and myeloid leukaemia (IARC, 2004)). A total of 1372 (8.3%) men had a psychiatric admission during follow-up; 431 (31.4%) of these had their first psychiatric admission after their cancer registration and were excluded from the main analysis. Men with psychiatric admissions were of lower socioeconomic status, less educated, more likely to have other comorbidities (based on hospital admissions for causes other than cancer, psychiatric disorders or suicide), and were more likely to smoke, have used illegal drugs, and engage in risky alcohol use. Smoking-related cancers were more common in men with a psychiatric hospital admission (*N*=299 (31.8%)) relative to men without (14.6%).

**Figure 1:**
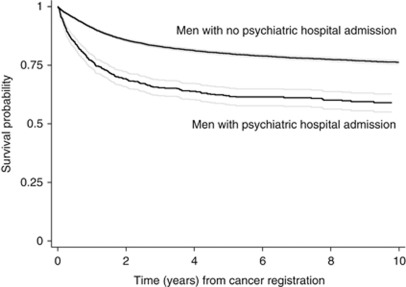
Kaplan–Meier survival times to cancer death from cancer registration in men with (*N*=941) and without (*N*=15 126) hospital admissions for psychiatric disorder. Follow-up started on the date of cancer registration and ended on the earliest of: date of death, date of emigration, or 31 December 2004, the last date on which deaths were available. Men whose cause of death was something other than cancer were censored on their date of death and did not contribute to cancer case-fatality rates.

Men with psychiatric admissions were, on average, 5.4 years older at the time of cancer registration than men without. One year after cancer registration, 215 (22.8%) men with a psychiatric admission had died from cancer compared with 1321 (8.7%) of men without. The corresponding results at 2 and 5 years were: 272 (28.9%) *vs* 2007 (13.3%), and 312 (33.2%) *vs* 2644 (17.5%). Ten-year survival from the date of cancer registration in men with and without psychiatric admissions is shown in Figure 1. Men with a psychiatric admission had markedly worse survival (*P*<0.001) in the years immediately following cancer registration. Once established, this differential did not diverge further in subsequent years.

Hazard ratios (HRs) (95% confidence interval (CI)) comparing cancer mortality in men with and without psychiatric admissions are shown in Table 1. Men with any psychiatric admission were more than twice as likely to die from their cancer as men without (multiply-adjusted HR (95% CI): 2.13 (1.86, 2.44)). Data on smoking, drug use, and alcohol were available for a subset (9.4%) of the cohort and adjustment for these factors did not alter the results. Additional adjustment for age at cancer registration somewhat attenuated the association (multiply- and age-adjusted HR (95% CI): 1.59 (1.39, 1.83)). Results for psychiatric subtypes were broadly similar, with the possible exception of bipolar disorders, for which just 36 men had admissions. The greatest case fatality was observed in men with admissions for depressive, alcohol, and other substance use disorders. Separate analyses of smoking and nonsmoking-related cancers were very similar. Analyses including men whose cancer pre-dated their psychiatric admission produced slightly weaker results (multiply- and age-adjusted HR (95% CI): 1.30 (1.15, 1.47)). In this case, men with psychiatric admissions were, on average, 2.7 years older at cancer registration.

## Discussion

Among male cancer patients, those who also had a psychiatric admission tended to be older at the time of cancer registration and more likely to die from cancer, particularly in the years immediately following registration, relative to those with no history of serious mental illness. We are only aware of one other study (Lawrence *et al*, 2000) that has directly investigated this association. This study, from Western Australia, reported 25% higher case fatality in male psychiatric patients.

The current analyses utilise an almost complete birth cohort of male cancer patients followed-up for over two decades. The large sample size offers superior statistical power and the longitudinal design allowed identification of incident psychiatric illness, cancer registrations, and deaths. Psychiatric disorders were identified from hospital admissions, guaranteeing clinically identified problems; cancers were reliably identified from registrations made, by law, by clinicians and pathologists, and from death certificates. We attempted to address potential reverse causality by excluding men whose first psychiatric admission occurred after their cancer registration. This approach has been used previously, but as the exact onset of both psychiatric disorder and cancer may be difficult to establish, this approach may be overly conservative in practice (Whitley *et al*, 2012). Results from analyses including these men, although weaker, remained consistent with poorer survival in men with psychiatric admissions, suggesting that observed associations were not solely a consequence of these exclusions.

There are also a number of limitations. The use of hospital discharge data limits our results to the impact of psychiatric problems severe enough to warrant a hospital admission. If individuals with less severe psychiatric disorders are less likely to exhibit poor health behaviours, or more likely to comply with cancer treatment than those with psychiatric hospitalisation, then our results may be over-estimates. Alternatively, a psychiatric hospitalisation may increase the chances of cancer diagnosis and treatment, and may also improve health behaviours, albeit temporarily, in which case associations presented here may be under-estimates. It is also important to note that hospitalisation rates will vary for different psychiatric subtypes and this may influence our results, particularly those for specific psychiatric diagnoses. In addition, the cohort consisted of young men at conscription. This is a strength in terms of identifying psychiatric problems, which are more common in younger individuals. However, in spite of lengthy follow-up, cohort members were aged 55 years or less at the end, which is relatively young in terms of cancer development, and we were unable to perform cancer site-specific analyses. Finally, our data are restricted to men born in Sweden from 1950 to 1976, which limits the generalisability; in particular, we cannot comment on cancer case fatality in women, which may be distinct from that in men.

There are a number of plausible explanations for our observations. For example, individuals with mental illness are more likely to have comorbidities additional to cancer, for example, cardiovascular disease or diabetes, which may impact on survival, although adjustment for comorbidity based on hospital admissions did not affect our results. Alternatively, evidence suggests that psychiatric patients are less able or willing to comply with cancer treatment and this may shorten their survival time (Howard *et al*, 2010). Psychiatric patients also have worse health behaviours than the general population (Davidson *et al*, 2001; McCreadie, 2003; Daumit *et al*, 2005; de Leon and Diaz, 2005) and, in addition to influencing cancer incidence, these may also affect case fatality. For example, obesity prevalence is higher in this group, although adjustment for BMI did not explain our results. Alternatively psychiatric patients are known to smoke more (de Leon and Diaz, 2005) and survival for some smoking-related cancers, most notably lung, is known to be particularly poor (Coleman *et al*, 2003). In our cohort, smoking-related cancers were more common in those with psychiatric hospital admissions. We did not have sufficient numbers to examine site-specific survival; however, comparable psychiatric admission-survival associations were evident in separate analyses of smoking and nonsmoking-related cancers, indicating that smoking differences alone do not explain our results.

It is also worth considering the role of delayed cancer diagnosis. Individuals with mental illness consult their physicians frequently but may have more contact with psychiatric staff who may be less skilled in diagnosing malignancies. Another concern is ‘diagnostic overshadowing’ whereby physical symptoms are ascribed to an existing psychiatric condition (Howard *et al*, 2010). It is therefore possible that psychiatric patients have cancers diagnosed later, and therefore at a more advanced stage, than the general population. Consistent with this, men in our cohort with psychiatric admissions were >5 years older at the time of their first cancer registration than men without, and survival in men with psychiatric admissions was particularly poor in years immediately following cancer registration. Adjustment for age at cancer diagnosis attenuated associations to some extent but not entirely, suggesting that age differences alone do not explain these associations.

## Figures and Tables

**Table 1:** Hazard ratio (95% confidence interval) for cancer deaths according to hospital admissions for psychiatric disorder

	**(Cancer death/no cancer death)**	**Multiply adjusted^a^**	**Additionally adjusted for age at cancer diagnosis**
*All psychiatric disorders*			
No admission	3010/12 116	1.00	1.00
1+ admission	322/619	2.13 (1.86, 2.44)	1.59 (1.39, 1.83)
*P*		<0.001	<0.001

*Schizophrenia* b			
No admission	3010/12 116	1.00	1.00
1+ admission	40/71	1.95 (1.31, 2.90)	1.49 (1.00, 2.23)
*P*		0.001	0.05

*Non-affective psychosis* c			
No admission	3010/12 116	1.00	1.00
1+ admission	33/66	1.73 (1.13, 2.64)	1.39 (0.91, 2.12)
*P*		0.01	0.13

*Bipolar disorders* d			
No admission	3010/12 116	1.00	1.00
1+ admission	9/27	1.32 (0.59, 2.96)	0.95 (0.43, 2.12)
*P*		0.49	0.90

*Depressive disorders* e			
No admission	3010/12 116	1.00	1.00
1+ admission	76/154	2.33 (1.73, 2.88)	1.59 (1.23, 2.06)
*P*		<0.001	<0.001

*Neurotic and adjustment disorders* f			
No admission	3010/12 116	1.00	1.00
1+ admission	82/205	1.82 (1.42, 2.32)	1.36 (1.06, 1.74)
*P*		<0.001	0.01

*Personality disorders* g			
No admission	3010/12 116	1.00	1.00
1+ admission	42/95	1.79 (1.26, 2.55)	1.35 (0.94, 1.92)
*P*		0.001	0.10

*Alcohol-related disorders* h			
No admission	3010/12 116	1.00	1.00
1+ admission	153/266	2.31 (1.92, 2.77)	1.66 (1.38, 2.00)
*P*		<0.001	<0.001

*Other substance use disorders* i			
No admission	3010/12 116	1.00	1.00
1+ admission	78/133	2.16 (1.67, 2.81)	1.62 (1.25, 2.11)
*P*		<0.001	<0.001

a Adjusted for socioeconomic status in childhood, socioeconomic status in adulthood, highest educational attainment, body mass index and comorbidities (based on hospital admissions for causes other than cancer, psychiatric disorders or suicide).

b ICD 8/9 codes: 295; ICD 10: F20–21, F25.

c ICD 8: 297, 298.2–3, 298.9; ICD 9: 297, 298.2–4, 298.8–9; ICD 10: F22–24, F28–29.

d ICD 8: 296.1, 296.3, 298.1; ICD 9: 296.0, 296.2–5, 298.1; ICD 10: F30–31.

e ICD 8: 296.0, 296.2, 298.0, 300.4; ICD 9: 296.1, 298.0, 300.4, 311; ICD 10: F32–34, F38–39.

f ICD 8: 300.0–3, 300.5–9, 305, 307; ICD 9: 300.0–3, 300.5–9, 306, 308–9; ICD 10: F40–48.

g ICD 8/9: 301; ICD 10: F60–69.

h ICD8: 291, 303; ICD 9: 291, 303, 305.0; ICD 10: F10.

i ICD8: 294.3, 304; ICD 9: 292, 304, 305.1–8; ICD 10: F11–F19.
